# Экспрессии микроРНК в плазме крови, оттекающей от гипофиза, у пациентов с болезнью Иценко-Кушинга и АКТГ-эктопированным синдромом

**DOI:** 10.14341/probl12817

**Published:** 2021-12-30

**Authors:** А. А. Малыгина, Ж. Е. Белая, А. Г. Никитин, Ф. А. Кошкин, И. И. Ситкин, А. М. Лапшина, П. М. Хандаева, А. С. Луценко, Д. А. Трухина, Г. А. Мельниченко

**Affiliations:** Национальный медицинский исследовательский центр эндокринологии; Национальный медицинский исследовательский центр эндокринологии; Научно-исследовательский институт пульмонологии; Медико-генетический центр «Геномед»; Национальный медицинский исследовательский центр эндокринологии; Национальный медицинский исследовательский центр эндокринологии; Национальный медицинский исследовательский центр эндокринологии; Национальный медицинский исследовательский центр эндокринологии; Национальный медицинский исследовательский центр эндокринологии; Национальный медицинский исследовательский центр эндокринологии

**Keywords:** Болезнь Иценко-Кушинга, АКТГ-эктопированный синдром, микроРНК, NGS, аденома гипофиза

## Abstract

**ОБОСНОВАНИЕ:**

ОБОСНОВАНИЕ.  За последние десятилетия микроРНК зарекомендовали себя как новые маркеры для целого ряда заболеваний. Определение специфичной панели микроРНК, отличающей пациентов с БИК и АКТГ-эктопированным синдромом (АКТГ-ЭС), смогло бы значительно упростить диагностику.

**ЦЕЛЬ:**

ЦЕЛЬ. Выявить циркулирующие микроРНК, отличающиеся у пациентов с БИК и АКТГ-ЭС в плазме крови, оттекающей от гипофиза.

**МАТЕРИАЛЫ И МЕТОДЫ:**

МАТЕРИАЛЫ И МЕТОДЫ. Выполнено одноцентровое, одномоментное, выборочное исследование случай-контроль. Включено 24 пациента с АКТГ-зависимым ЭГ, которым требовалось проведение селективного забора крови из нижних каменистых синусов (НКС). Из них 12 пациентов с БИК (м=2, ж=10), возрастная медиана 46,5 лет [33,8;53,5] и 12 пациентов с АКТГ-ЭС (м=4, ж=8), возрастная медиана 54 года [38,75;60,75].  Образцы плазмы крови из НКС были получены до введения стимуляционного агента, центрифугированы и заморожены при температуре -80С. Выделение микроРНК из плазмы крови проводили с помощью miRNeasy Serum/Plasma Kit («Qiagen», Германия) согласно инструкции компании-производителя на автоматической станции QIAcube («Qiagen», Германия). Концентрацию суммарной РНК в водном растворе оценивали на спектрофотометре NanoVue Plus («GE Healthcare», Великобритания). Библиотеки были подготовлены с помощью QIAseq miRNA Library Kit согласно стандартным протоколам производителя. Экспрессию микроРНК исследовали с помощью секвенирования на Illumina NextSeq 500 (Illumina NextSeq 500, США).

**РЕЗУЛЬТАТЫ:**

РЕЗУЛЬТАТЫ. Обнаружены 108 дифференциально экспрессирующихся микроРНК  (p <0,05) после поправки на множественность сравнений. МикроРНК разделены на три группы – группа 1 с числом прочтений более 10 в обеих группах сравнения, группа 2 с числом прочтений менее 10 в одной из групп, группа 3 с числом прочтений менее 10 в обеих группах. Для верификации методом количественной ПЦР с обратной транскрипцией (RT-qPCR), планируется использовать следующие микроРНК: miR-383-3p, miR-4290 и miR-6717-5p, экспрессия которых была повышена у пациентов с БИК по сравнению с пациентами из группы АКТГ-ЭС в 46,36 раз (p=0,01), в 6,84 раз (p=0,036) и в 4,49 раз (p=0,031), соответственно,  miR-1203, miR-1229-3p, miR-639, экспрессия которых была снижена у пациентов с БИК в 36,74 раз (p=0,013), в 78,3 раз (p=0,003), в 73,22 раза (p=0,002), соответственно, а также miR-302c-3p,  экспрессия которой была повышена у пациентов с БИК в 92,69 раз (p=0,001).

**ЗАКЛЮЧЕНИЕ:**

ЗАКЛЮЧЕНИЕ. В ходе исследования был значительно расширен список микроРНК-кандидатов для диагностики АКТГ-зависимых форм ЭГ. Необходима валидизация полученных микроРНК в периферической крови на расширенной выборке пациентов методом RT-qPCR.

## Abstract (en)

**BACKGROUND:**

BACKGROUND: For the last decades microRNAs (miR) have proven themselves as novel biomarkers for various types of diseases. Identification of specific circulating microRNA panel that differ patient with Cushing’s disease (CD) and ectopic ACTH syndrome (EAS) could improve the diagnostic procedure.

**AIM:**

AIM: to evaluate the differences in miR levels in plasma samples drained from inferior petrosal sinuses in patients with CD and EAS.

**MATERIALS AND METHODS:**

MATERIALS AND METHODS: single-center, case-control study: we enrolled 24 patients with ACTH-dependent Cushing’s syndrome (CS) requiring bilateral inferior petrosal sinus sampling (BIPSS).  Among them 12 subjects were confirmed as CD (males=2, females=10; median age 46,5 [IR 33,8;53,5]) and 12 as EAS (males=4, females=8, median age 54 [IR 38,75;60,75]). BIPSS was performed through a percutaneous bilateral approach. Once catheters were properly placed, blood samples were withdrawn simultaneously from each petrosal sinus and a peripheral vein. Plasma samples from both sinuses were centrifuged and then stored at -80 C. MiRNA isolation from plasma was carried out by an miRneasy Plasma/Serum Kit (Qiagen, Germany) on the automatic QIAcube station according to the manufacturer protocol. To prevent degradation, we added 1 unit of RiboLock Rnase Inhibitor (Thermo Fisher Scientific, USA) per 1 μL of RNA solution. The concentration of total RNA in the aqueous solution was evaluated on a NanoVue Plus spectrophotometer (GE Healthcare, USA). The libraries were prepared by the QIAseq miRNA Library Kit following the manufacturer standard protocols. MiR expression was then analyzed by sequencing on Illumina NextSeq 500 (Illumina, USA).

**RESULTS:**

RESULTS: 108 miRNAs were differently expressed (p <0,05) in inferior petrosal sinus samples of patients with CD vs EAS. We divided these miRNAs into 3 groups based on the significance of the results. The first group consisted of samples with the highest levels of detected miR in both groups. Four miRNAs were included: miR-1203 was downregulated in CD vs EAS — 36.74 (p=0,013), and three other were upregulated in CD vs EAS: miR-383-3p 46.36 (p=0,01), miR-4290 6.84 (p=0,036), miR-6717-5p 4.49 (p=0,031). This miRs will be validated in larger cohorts using RT-qPCR.

**CONCLUSION:**

CONCLUSION: Plasma miR levels differ in inferior petrosal samples taken from patients with CD vs EAS. These miRs need to be validated by different methods and in peripheral plasma samples in order to be used as potentially non-invasive biomarkers to differentiate ACTH-dependent CS.

## ОБОСНОВАНИЕ

Болезнь Иценко–Кушинга (БИК) — тяжелое нейроэндокринное заболевание, сопровождающееся развитием серьезных инвалидизирующих осложнений и повышенной смертностью [1]. БИК является наиболее частой причиной эндогенного гиперкортицизма (ЭГ) (до 70–80% случаев) [2], характеризуется гиперкортизолемией, развившейся вследствие гиперпродукции адренокортикотропного гормона (АКТГ) кортикотропиномой гипофиза [3]. Женщины болеют чаще мужчин в соотношении 3–4:1 [4]. Другой АКТГ-зависимой формой ЭГ является АКТГ-эктопированный синдром (АКТГ-ЭС) (5–10%) [5], когда симптомокомплекс гиперкортицизма развивается вследствие паранеоплаcтического синдрома и источник гиперпродукции АКТГ находится в других органах и тканях — в легких, тимусе, щитовидной железе, поджелудочной железе, толстом кишечнике и др. [6]. При этом приблизительно в 19% случаев АКТГ-ЭС локализовать опухоль не удается [7]. АКТГ-ЭС одинаково распространен как среди мужчин, так и среди женщин [7]. Клиническая картина БИК и АКТГ-ЭС обусловлена в первую очередь проявлениями хронической гиперкортизолемии и характеризуется перераспределением подкожножировой клетчатки, повышением артериального давления, нарушением углеводного обмена, образованием характерных багровых стрий на поверхности передней брюшной стенки, груди, плеч, матронизмом, мышечной слабостью, патологическими переломами костей скелета, ломкостью капилляров (приводящей к легкому образованию кровоизлияний, экхимозов и петехий), нарушением менструальной функции у женщин, снижением потенции у мужчин [8, 9]. Несмотря на то что причины гиперпродукции АКТГ разные и тактики лечения, соответственно, тоже, установить диагноз по сумме клинических проявлений и комплекса лабораторно-инструментальных исследований не всегда представляется возможным. В связи с этим ряд пациентов за все годы активного лечения АКТГ-зависимого ЭГ проходили через заведомо неэффективные вмешательства на гипофизе в виде транссфеноидальной аденомэктомии или радиохирургии [10]. Причинами этого являются несовершенство имеющихся в арсенале врача методов лабораторной диагностики, отсутствие высокочувствительных тестов для дифференциальной диагностики АКТГ-зависимых форм ЭГ [11]. Несмотря на то что разрабатываются неинвазивные алгоритмы дифференциальной диагностики из имеющихся тестов и проб, золотым стандартом остается селективный забор крови из нижних каменистых синусов (НКС) [12][13]. Ценность селективного забора крови из НКС неоспорима, однако данная процедура инвазивна и требует оснащенной рентген-операционной и высокой квалификации сосудистого хирурга. Чувствительность и специфичность селективного забора крови варьируют в пределах 88–100 и 67–100 соответственно [14]. Такая разница в показателях эффективности частично объясняется разной методологией проведения процедуры (наличие/отсутствие стимулирующего агента) и опытностью хирургов. Критически важным этапом, влияющим на ценность результата всей процедуры, является правильное позиционирование катетеров в НКС, для подтверждения чего предложено использовать определение градиента пролактина до введения стимулирующего агента [15]. Тем не менее такой контроль проводится не всегда, отчего повышаются шансы ошибочного заключения. К наиболее частым побочным эффектам относится гематома в области венепункции, тем не менее зарегистрированы случаи и более серьезных осложнений, таких как перфорации бедренной вены, формирование артериовенозной фистулы, тромбоз глубоких вен, тромбоэмболии легочной артерии и даже случай летального исхода в результате тромбоза кавернозного синуса [16]. Учитывая трудоемкость селективного забора, для улучшения дифференциальной диагностики АКТГ-зависимого ЭГ необходимы более доступные и неинвазивные методы.

МикроРНК — класс малых (~23 нуклеотида) некодирующих молекул РНК, играющих важную роль в посттранскрипционной регуляции генов через связывание с матричной РНК (мРНК). Как правило, связь микроРНК с мРНК приводит к ее деградации или препятствует считыванию белок-кодирующей последовательности [17]. Первые микроРНК были открыты более 20 лет назад, однако прошло более 10 лет, прежде чем эти молекулы из биологии начали «приходить» в медицинские исследования [18–22]. Будучи достаточно стабильными циркулирующими молекулами, концентрацию которых можно измерить в разных биологических жидкостях, микроРНК могут служить надежными онкомаркерами [23]. В 2002 г. было впервые описано влияние измененного уровня экспрессии конкретных микроРНК на развитие онкологического заболевания — сниженный уровень экспрессии miR-15 и miR-16, возникающий при делеции 13q14.3, приводит к развитию хронического лимфолейкоза [24]. Далее последовал целый ряд исследований по поиску изменений в экспрессии микроРНК, характерных для различных неоплазий, и на данный момент найдены специфические изменения профилей микроРНК при раке простаты [25], молочной железы [26], шейки матки [27], легких [28] и др.

В 2015 г. на базе ФГБУ ЭНЦ Минздрава России было выполнено исследование уровня экспрессии ранее описанных в литературе микроРНК в плазме крови пациентов с АКТГ-зависимым ЭГ [29]. В исследовании участвовал 41 пациент с АКТГ-зависимым ЭГ (n=28 БИК, n=13 АКТГ-ЭС) и 11 человек из группы контроля. У всех участников выполнен забор плазмы крови натощак, проведен анализ 21 микроРНК (miR-10b-5p, miR-129-5p, miR-133a-5p, miR-141-3p, miR-143-3p, miR-145-5p, miR-150-3p, miR-15a-5p, miR-16-5p, miR-146a-5p, miR-185-3p, miR-191-5p, miR-203a-5p, miR-210-5p, miR-211-5p, miR-31-5p, miR-409-3p, miR-409-5p, miR-431-5p, miR-488-3p, miR-7g-5p) методом количественной полимеразной цепной реакции с обратной транскрипцией (RT-qPCR). Статистически значимая разница уровня экспрессии между пациентами с БИК и АКТГ-ЭС была обнаружена по уровням miR-16-5p [ 45,04 (95% доверительный интервал (ДИ) 28,77–61,31) у пациентов с БИК vs. 5,26 (95% ДИ 2,65–7,87) в группе АКТГ-ЭС, p<0,001; q=0,001], miR-145-5p [0,097 (95% ДИ 0,027–0,167) у пациентов с БИК vs. неопределяемый уровень у пациентов с АКТГ-ЭС, p=0,008; q=0,087] и miR-7g-5p [ 1,842 (95% ДИ 1,283–2,400) у пациентов в БИК vs. 0,847 (95% ДИ 0,187–1,507) в группе АКТГ-ЭС, p=0,02; q=0,14]. Таким образом, было выявлено 3 микроРНК, статистически значимо отличающихся у пациентов с БИК и АКТГ-ЭС, при этом наибольшие различия показала miR-16-5p, уровень экспрессии которой отличался не только между пациентами с разным генезом АКТГ-зависимого ЭГ, но также между пациентами с разными формами АКТГ-зависимого ЭГ и здоровыми добровольцами.

Планировалось провести анализ экспрессии микроРНК в крови, оттекающей от гипофиза, у пациентов с разными формами АКТГ-зависимого гиперкортицизма методом высокопроизводительного секвенирования в целях выявления большего количества микроРНК, потенциально пригодных для формирования панели для дифференциальной диагностики АКТГ-зависимых форм ЭГ.

## ЦЕЛЬ ИССЛЕДОВАНИЯ

Выявить различно экспрессирующиеся циркулирующие микроРНК в плазме крови из НКС у пациентов с БИК и АКТГ-ЭС.

## МАТЕРИАЛЫ И МЕТОДЫ

Место и время проведения исследования

Место проведения. Исследование проведено на базе отделения нейроэндокринологии и остеопатий ФГБУ «НМИЦ эндокринологии» Минздрава России. Высокопроизводительное секвенирование выполнено на базе лаборатории «Геномед». Выделение микроРНК и биоинформатический анализ полученных данных выполнены на базе ФГБУ «НИИ пульмонологии» ФМБА России.

Время исследования. Набор пациентов осуществлялся с сентября 2017 г. по май 2019 г.

Изучаемые популяции

В исследование были включены пациенты с подтвержденным диагнозом АКТГ-зависимого гиперкортицизма, поступившие в отделение нейроэндокринологии и остеопатий ФГБУ «НМИЦ эндокринологии» Минздрава России для проведения селективного забора крови из НКС.

Критерии включения: АКТГ-зависимый гиперкортицизм, подтвержденный на основании критериев, установленных действующими клиническими рекомендациями по диагностике БИК; аденома гипофиза менее 6 мм в диаметре, или отсутствие визуализации аденомы по данным МРТ, или неэффективная транссфеноидальная аденомэктомия в анамнезе при отсутствии данных гистологического исследования послеоперационного материала.

Критерии исключения: возраст моложе 18 лет, прием препаратов, снижающих уровень кортизола, тяжелые системные заболевания, беременность, множественные метастатические поражения у пациентов с АКТГ-ЭС.

Дизайн исследования

Проведено одноцентровое одномоментное выборочное исследование случай-контроль.

Описание медицинского вмешательства (для интервенционных исследований)

Всем пациентам выполнена процедура селективного забора крови из НКС. Доступ осуществлялся через бедренную вену, через которую под рентгенологическим контролем после установки интродьюсеров 4F и 5F проводились многоцелевые катетеры 4F, которые устанавливались в правом и левом НКС. До введения стимуляционного агента осуществлялся забор плазмы крови из НКС в пробирки с этилендиаминтетраацетатом (ЭДТА). Далее проводились двукратное центрифугирование образцов (лабораторная центрифуга Eppendorf 5810R с комплектом роторов (А-4-81, Ф-4-81-MTP/Flex, FA-45-30-11 и F-45-48-PCR)) на скорости 3000 оборотов в минуту в течение 15 мин при температуре +5оС, забор плазмы и замораживание материала при температуре -80 оС до дальнейшего анализа.

Методы

Перед проведением селективного забора крови из НКС все пациенты проходили лабораторное обследование для подтверждения активности гиперкортицизма: выполнялись анализ слюны на кортизол в 23:00 на автоматическом анализаторе Cobas е601 фирмы F. Hoffmann-LaRocheLtd (каталожный № 11875116 122), используя метод электрохемилюминесцентного анализа (ЭХЛА) (точка разделения 9,4 нмоль/л) [30], сбор суточной мочи с определением свободного кортизола иммунохемилюминесцентным методом на аппарате Vitros ECi с предварительной экстракцией диэтиловым эфиром (референсный интервал 60–413 нмоль/сут), малая проба с 1 мг дексаметазона (точка разделения более 50 нмоль/л), для оценки генеза ЭГ выполнялся анализ крови на АКТГ утром (референсный интервал 7–66 пг/мл), значение кортизола утром, равное или более 10 пг/мл, указывало на центральный генез ЭГ. Для оценки циркадного ритма выработки АКТГ оценивался его уровень в 23:00 (референсный интервал 0–30 пг/мл). Исследование уровней кортизола и АКТГ плазмы крови выполнено на электрохемилюминесцентных анализаторах фирмы Roche (Elecsys 2010; Cobas e601) стандартными наборами фирмы F. Hoffmann-La Roche Ltd.

Всем пациентам была выполнена МРТ гипофиза в целях поиска аденомы на магнитно-резонансном томографе Magnetom Harmony (Siemens, Германия) с введением контрастного вещества по показаниям.

Выделение микроРНК из плазмы крови проводили с помощью miRNeasy Serum/Plasma Kit (Qiagen, Германия) согласно инструкции компании-производителя на автоматической станции QIAcube (Qiagen, Германия). Для предотвращения деградации в выделенную РНК добавляли 1 ед. RiboLock RNase Inhibitor (Thermo Fisher Scientifi», США) на 1 мкл раствора нуклеиновых кислот. Концентрацию суммарной РНК в водном растворе оценивали на спектрофотометре NanoVue Plus (GE Healthcare, Великобритания). Для дальнейшей работы отбирали образцы с концентрацией суммарной РНК в водном растворе не ниже 5 нг/мкл. Экспрессию микроРНК анализировали с помощью секвенирования на Illumina NextSeq 500 (Illumina NextSeq 500, США). Библиотеки были подготовлены с помощью QIAseq miRNA Library Kit в соответствии со стандартными протоколами производителя. Контроль качества библиотек выполнялся на Lab Chip GX. Биоинформационная обработка была следующей: адаптеры удалялись с помощью Cutadapt; полученные файлы FASTQ были затем картированы на геном человека (сборка GRCh37) с помощью bowtie2. FastQC использовался в качестве инструмента для визуализации различных измерений контроля качества. Для каждого образца последовательности аннотировалась с использованием баз данных человеческих пре-микроРНК и зрелых микроРНК, предоставленных в miRBase (http://microrna.sanger.ac.uk/sequences/) с помощью SeqBuster.TargetScan, Diana-TarBase v8 и mirPath v.3, были использованы для предсказания мишеней. Платформа miRNet использовалась для анализа взаимодействия микроРНК и их генов-мишеней.

Статистический анализ

Размер выборки предварительно не рассчитывался ввиду редкости заболевания. Основные количественные характеристики пациентов представлены в виде медианы и интерквартильного размаха. Сравнение описательных параметров пациентов с БИК и АКТГ-ЭС выполнено с использованием непарных двусторонних t-тестов. Для сравнения качественных параметров двух независимых групп использован точный критерий Фишера. Статистически значимым считалось значение p<0,05. Аналитическая статистика выполнена с помощью статистического пакета SPSS 23.0. Биоинформатический анализ данных секвенирования выполнен при помощи пакета DESeq2.

Этическая экспертиза

Проведение исследования было одобрено комитетом по этике ФГБУ «Эндокринологический научный центр» Минздрава России (протокол №1 от 25.01.2017 г.) в рамках темы «Постгеномные технологии и неинвазивные методы в диагностике эндогенного гиперкортицизма и его АКТГ-зависимых форм».

## РЕЗУЛЬТАТЫ

Объекты (участники) исследования

В исследование включены 12 пациентов с диагнозом БИК и 12 пациентов с диагнозом АКТГ-ЭС. Исходные характеристики участников исследования сведены в таблице 1. В группе пациентов с БИК у 3 (25%) не было выявлено аденомы гипофиза по данным МРТ, у 9 пациентов (75%) по данным МРТ было выявлено образование гипофиза менее 6 мм, в связи с чем и был выполнен селективный забор крови из НКС. В когорте пациентов с АКТГ-ЭС у 7 пациентов (58,3%) была обнаружена нейроэндокринная опухоль (НЭО) легкого, у 1 пациента (8,3%) — НЭО правого надпочечника, у 1 пациента (8,3%) — НЭО поджелудочной железы, у 3 пациентов (24,9%) источник эктопической продукции АКТГ не идентифицирован. Окончательный диагноз устанавливался на основании клинической и лабораторной ремиссии после операции, а также по данным иммуногистохимического (ИГХ) исследования послеоперационного материала. Пациенты, у которых в результате селективного забора крови из НКС был подтвержден центральный генез ЭГ, были направлены на нейрохирургическое лечение в отделение нейрохирургии ФГБУ «НМИЦ эндокринологии» Минздрава России. У 11 из 12 пациентов зарегистрирована послеоперационная ремиссия, у 1 пациентки после выполнения повторной транссфеноидальной аденомэктомии ремиссии достигнуто не было, учитывая тяжесть состояния было принято решение о проведении двусторонней адреналэктомии. ИГХ-исследование было выполнено на 7 образцах послеоперационного материала: в 3 случаях выявлена плотногранулированная кортикотропинома, еще в 3 случаях — редкогранулированная кортикотропинома, в одном случае опухолевой ткани выявлено не было (табл. 2).

**Table:** Таблица 1. Сравнительная характеристика участников исследования БИК — болезнь Иценко-Кушинга, АКТГ-ЭС — АКТГ-эктопированный синдром, ИМТ — индекс массы тела, АКТГ — адренокортикотропный гормон, P — уровень значимости, n — количество пациентов, м — мужчины, ж — женщины.

Параметры	БИК	АКТГ-ЭС	P
Количество	12	12	
Возраст, годы	46,5 [ 33,8; 53,5]	54 [ 38,75; 60,75]	0,343
Пол (м/ж)	2/10	4/8	0,515
ИМТ, кг/м2	30,7 [ 29,8; 36,2]	33,4 [ 24,6; 41,4]	0,987
АКТГ крови в 23:00, пг/мл	51,3 [ 35; 73,9]	112,8 [ 66,3; 193,7]	0,007

**Table:** Таблица 2. Результаты иммуногистохимического исследования послеоперационного материала пациентов с болезнью Иценко–Кушинга ПГК — плотногранулированная кортикотропинома, РГК — редкогранулированная кортикотропинома.

№	ИГХ
1	ПГК
2	РГК
3	РГК
4	РГК
5	ПГК
6	ПГК
7	При дорезке с блока в срезах, окрашенных гематоксилином и эозином, импрегнацией серебром и ИГХ с антителами к АКТГ опухолевой ткани не обнаружено.

Основные результаты исследования

Были обнаружены 108 дифференциально экспрессирующихся микроРНК с уровнем значимости <0,05 после поправки на множественность сравнений. По достоверности обнаруженных различий микроРНК разделены на три группы — группа 1 с числом прочтений более 10 в обеих группах сравнения (табл. 3), группа 2 с числом прочтений менее 10 в одной из групп, группа 3 с числом прочтений менее 10 в обеих группах (табл. 4 и 5). Для групп 2 и 3 из-за низкой представленности микроРНК потребуется большее количество образцов в группах при валидации обнаруженных изменений с помощью RT-qPCR. Тепловая карта экспрессии 108 микроРНК в группе пациентов с БИК по сравнению с АКТГ-ЭС представлена на рис. 1.

**Table:** Таблица 3. МикроРНК с числом прочтений более 10 для обеих групп сравнения (группа 1)

микроРНК	Изменение экспрессии	Уровень значимости p
hsa-miR-1203	-36,74	0,013
hsa-miR-383-3p	46,36	0,01
hsa-miR-4290	6,84	0,036
hsa-miR-6717-5p	4,49	0,031

**Table:** Таблица 4. МикроРНК с числом прочтений менее 10 в одной из групп (группа 2)

miR-1185-2-3p	26,23	0,038
miR-1229-3p	-78,3	0,003
miR-1238-5p	22,61	0,05
miR-1265	85,85	0,003
miR-1267	31,39	0,012
miR-1294	-21,65	0,036
miR-138-5p	30,44	0,005
miR-15b-3p	-14,29	0,036
miR-2114-3p	23,7	0,044
miR-2116-5p	35,37	0,022
miR-302c-3p	92,69	0,001
miR-31-5p	30,89	0,029
miR-3145-5p	32,13	0,027
miR-335-5p	36,81	0,012
miR-3683	-49,02	0,007
miR-3692-3p	-33,5	0,015
miR-376b-5p	32,52	0,021
miR-3928-3p	44,64	0,002
miR-3939	11,06	0,04
miR-4287	-43,19	0,013
miR-4427	-20,01	0,019
miR-4470	-60,35	0,005
miR-4476	24,31	0,042
miR-4516	11,34	0,045
miR-4632-3p	-42,63	0,011
miR-4693-5p	176,66	0,0001
miR-4752	72,89	0,006
miR-4771	24,24	0,036
miR-4792	33,77	0,014
miR-4804-5p	106,51	0,001
miR-504-5p	28,22	0,027
miR-505-5p	-48,24	0,004
miR-5100	56,83	0,001
miR-519e-5p	96,19	0,001
miR-521	-34,78	0,014
miR-532-5p	79,29	0,002
miR-548ay-3p	98,87	0,001
miR-548w	93,25	0,001
miR-555	23,77	0,044
miR-5584-3p	90,89	0,001
miR-5702	168,27	8,268
miR-613	-22,19	0,048
miR-639	-73,22	0,002
miR-6508-5p	50,38	0,009
miR-6735-5p	80,64	0,002
miR-6739-5p	28,9	0,014
miR-6784-5p	58,52	0,006
miR-6790-3p	-19,75	0,032
miR-6796-5p	23,64	0,008
miR-6802-5p	16,47	0,017
miR-6808-5p	-17,86	0,032
miR-6866-5p	116,55	0,001
miR-6879-5p	16,16	0,025
miR-6881-3p	44,87	0,001
miR-6881-5p	13,84	0,029
miR-7110-3p	36,64	0,012
miR-7111-3p	113,64	0,001
miR-7975	71,63	0,003
miR-8073	-25,49	0,033
miR-8088	20,53	0,049
miR-877-3p	-9,97	0,049
miR-877-5p	-20,45	0,048
miR-888-3p	31,28	0,028

**Table:** Таблица 5. МикроРНК с числом прочтений менее 10 в обеих группах (группа 3)

miR-1228-3p	66,25	0,003
miR-1252-5p	-24,28	0,029
miR-145-5p	32,67	0,026
miR-16-1-3p	32,67	0,026
miR-21-3p	22,34	0,048
miR-212-5p	26,89	0,036
miR-296-5p	33,91	0,019
miR-298	60,3	0,004
miR-29b-1-5p	42,09	0,013
miR-3125	-33,83	0,009
miR-3202	28,24	0,033
miR-331-5p	23,23	0,039
miR-3922-5p	27,93	0,034
miR-4481	54,11	0,005
miR-4498	26,22	0,038
miR-4645-3p	28,43	0,033
miR-4685-3p	38,67	0,005
miR-4762-3p	65,2	0,004
miR-4773	-27,69	0,029
miR-5003-5p	21,42	0,027
miR-5007-3p	17,35	0,05
miR-513b-5p	29,34	0,031
miR-5583-5p	-21,76	0,049
miR-5586-5p	39,09	0,015
miR-5699-3p	48,2	0,01
miR-6507-3p	13,95	0,036
miR-6726-5p	24,48	0,042
miR-6733-5p	27,86	0,034
miR-6739-3p	23,53	0,045
miR-6755-5p	22,5	0,048
miR-6792-5p	21,92	0,034
miR-6805-3p	26,72	0,03
miR-6815-5p	28,28	0,033
miR-6828-3p	29,92	0,03
miR-6846-3p	32,55	0,026
miR-6869-3p	27,96	0,028
miR-6878-3p	59,79	0,003
miR-718	29,96	0,019
miR-8063	21,82	0,044
miR-921	29,28	0,026
miR-93-3p	22,17	0,049

**Figure:**
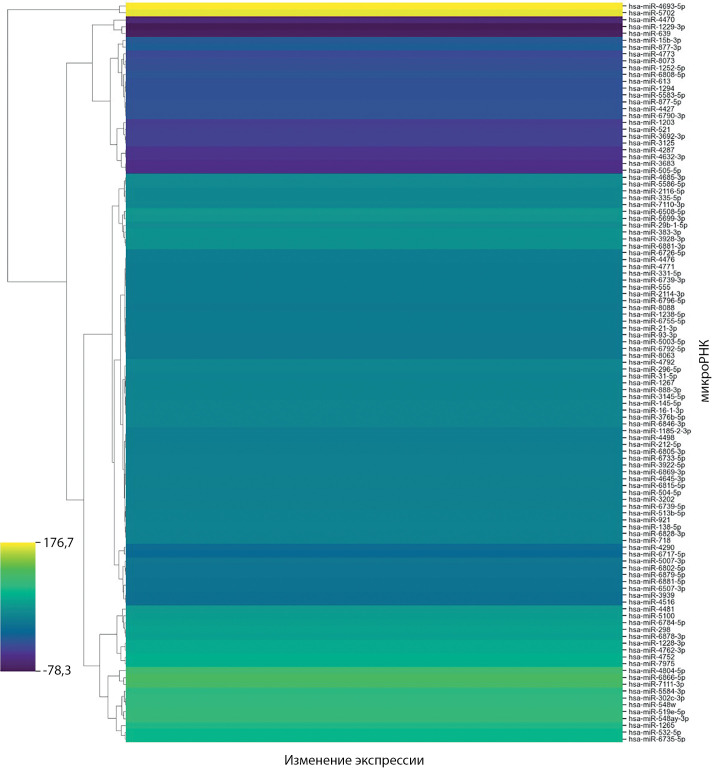
Рисунок 1. Тепловая карта экспрессии 108 микроРНК в группе пациентов с болезнью Иценко–Кушинга относительно пациентов с АКТГ-эктопированным синдромом. Синий цвет соответствует низкому уровню экспрессии, желтый — высокому.

Для дальнейшей верификации методом RT-qPCR планируется использовать следующие наиболее отличающиеся по профилю экспрессии между 2 группами микроРНК: miR-383-3p, miR-4290 и miR-6717-5p, экспрессия которых была повышена в группе пациентов с БИК по сравнению с пациентами из группы АКТГ-ЭС в 46,36 (p=0,01), 6,84 (p=0,03) и 4,49 раза (p=0,03) соответственно, miR-1203, miR-1229-3p, miR-639, уровень экспрессии которых был снижен у пациентов с БИК по сравнению с пациентами из группы АКТГ-ЭС в 36,74 (p=0,01), 78,3 (p=0,003), 73,22 раза (p=0,002) соответственно, а также miR-302c-3p, уровень экспрессии которой был повышен в группе пациентов с БИК по сравнению с АКТГ-ЭС в 92,69 раза (p=0,001).

Проведенный с помощью mirNet 2.0 [31] анализ показал, что обнаруженные микроРНК регулируют транскрипционные факторы и гены-онкосупрессоры (рис. 2). Значительная часть дифференциально экспрессирующихся микроРНК, которые мы обнаружили на первом этапе исследования, связана преимущественно с процессами онкогенеза и различными сигнальными путями, а также процессами передачи сигналов с помощью фокальных контактов. Значительное снижение экспрессии онкосупрессорных miR-302c-3p и miR-383-3p в АКТГ-ЭС может являться следствием происходящих в организме опухолевых процессов.

Нежелательные явления

За время проведения исследования нежелательные события не фиксировались.

## ОБСУЖДЕНИЕ

Сопоставление с другими публикациями

В данном исследовании мы продолжаем поиск различающихся по профилю экспрессии микроРНК у пациентов с БИК и АКТГ-ЭС. Забор крови, оттекающей от гипофиза, осуществлялся непосредственно в ходе проведения селективного забора крови из НКС. Нам удалось выявить порядка 108 микроРНК, которые различно экспрессировались в плазме крови, оттекающей от гипофиза, у пациентов с АКТГ-секретирующей аденомой (БИК) по сравнению со здоровым гипофизом у пациентов с АКТГ-ЭС.

Спустя сравнительно небольшое время после открытия микроРНК их начали изучать в отношении диагностики и дифференциальной диагностики образований гипофиза. Первая публикация результатов исследования относится к 2005 г., когда были впервые описаны изменения микроРНК в ткани аденом гипофиза на основании исследования 10 образцов соматотропином и 10 пролактином методом нозерн блоттинга. Была выявлена гипоэкспрессия miR-15a и miR16-1 в тканях аденом по сравнению с тканью здорового гипофиза [32–34]. По данным нашего исследования, miR-16-1-3p значится в группе 3 с числом прочтений менее 10 в обеих группах сравнения, при этом экспрессия данной микроРНК была в 32,7 раза выше у пациентов с БИК по сравнению с группой пациентов с АКТГ-ЭС (p=0,03). В 2009 г. были опубликованы первые результаты исследования экспрессии микроРНК в тканях кортикотропином при сравнении со здоровой тканью гипофиза [35]. При анализе данных 14 пациентов с БИК методом RT-qPCR в реальном времени было выявлено снижение уровня экспрессии let-7a, miR-15a, miR-16, miR-21, miR-141, miR-143, miR-145 и miR-150. По данным нашего исследования, miR-145-5p находится в группе 3 с количеством прочтений менее 10 в обеих группах сравнения, и уровень ее экспрессии был увеличен в группе пациентов с БИК в 32,7 раза (p=0,03), что подтверждает данные, полученные в пилотном исследовании [29], где уровень экспрессии miR-145-5p был также повышен в группе пациентов с БИК. В исследовании, опубликованном в 2010 г., было выявлено повышение экспрессии miR-122 и miR-493 в тканях АКТГ-продуцирующих карцином гипофиза при сравнении с тканью кортикотропином и здоровым гипофизом, что потенциально делает их маркерами злокачественности образования [36]. В 2021 г. была опубликована работа, в которой было выполнено сравнение профилей экспрессии микроРНК у пациентов с БИК, кортикостеромой надпочечника и здоровым контролем. Были проведены высокопроизводительное секвенирование образцов сыворотки пациентов, а также валидизация полученных микроРНК методом количественной ПЦР. Выявлено, что miR-182-5p значимо отличалась у пациентов с БИК по сравнению с кортикостеромой надпочечника и группой контроля [37]. В исследовании B. Lu и соавт. изучались взаимосвязь miR-16 и тенденции аденом гипофиза к инвазии в прилежащие ткани и усиленному росту. Снижение уровня экспрессии miR-16 было ассоциировано с более интенсивным ростом и склонностью к инвазии опухоли, предположительно через усиление выработки васкулярного эндотелиального фактора роста (VEGF2) через VEGFR2/p38/NF-κB сигнальный путь [38]. В исследовании W. Renjie и соавт. также выделяется роль miR-16 в подавлении миграции, пролиферации и инвазии клеток опухолей гипофиза наравне с miR-15a и miR-132, мишенью которых является Sox5 [39]. По данным F. Garbicz и соавт., экспрессии miR-93-3p, miR-25-3p, miR-93-5p и miR-106b-5p, кодируемые в 13м интроне гена MCM7, были значительно повышены в тканях инвазивных АКТГ-секретирующих аденом гипофиза, что делает данную панель потенциальным маркером инвазивного характера роста аденомы и фактором риска послеоперационных рецидивов и отсутствия ремиссии у пациентов после нейрохирургического вмешательства по поводу БИК [40].

При помощи базы данных TargetScan был выполнен анализ возможных генов-мишеней выявленных нами микроРНК (табл. 6). В TargetScan oценивается вероятность взаимодействия микроРНК и гена-мишени при помощи индекса cumulative weighted context++ score на основании 14 показателей вероятности связывания, при этом чем ближе значение к -3, тем больше вероятность воздействия определенной микроРНК на ген. В первую очередь был выполнен поиск связи выявленных микроРНК с описанными в литературе генами, изменения в которых были выявлены у пациентов с БИК и АКТГ-ЭС [41][42]. Следует отметить, что в базе данных TargetScan не содержится информации о miR-302c-3p. В результате поиска среди возможных мишеней числится USP8 (miR-383-3p), кодирующий убиквитин-специфическую протеазу 8. По разным данным, от 31 до 61% всех соматических мутаций, обнаруженных в кортикотропиномах, приходится на активирующие мутации гена USP8 [42]. При таких мутациях предотвращается деградация рецепторов к эпидермальному ростовому фактору (EGF) и стимулируется синтез POMC. Выявлена зависимость данного вида мутации от развития микроаденом гипофиза с высокой АКТГ-секретирующей активностью и склонностью к рецидивированию [43]. На момент написания данной статьи из всех исследованных генетических механизмов формирования кортикотропином мутация гена USP8 является одной из наиболее изученных и доказанных [44]. Описания других возможных генов-мишеней представлены в таблице 7.

**Table:** Таблица 6. Некоторые возможные мишени микроРНК по данным базы TargetScan

Общее количество генов-мишеней	miR-383-3p	miR-4290	miR-639	miR-6717-5p	miR-1203	miR-1229-3p
5483	3978	814	642	1058	4005
USP8	-0,18					
PRKAR1A		-0,01				-0,09
CABLES1	-0,07	0		-0,01		0
ZNF264	-0,07	-0,07				-0,09
GATA6	-0,03	-0,12				-0,01
CDKN1A	-0,14	-0,14	-0,39			
CDKN1B		-0,46				
TSC1					-0,29	-0,07
SSTR2						0
SSTR3	-0,02	-0,48				
SSTR5	-0,01	-0,32				
TP53					-0,13	
VHL	-0,33					-0,29
SDHC	-0,14					-0,22
MAX		-0,17			-0,59	
TMEM127	-0,03				-0,13	
SOX4		-0,23			-0,3	

**Figure:**
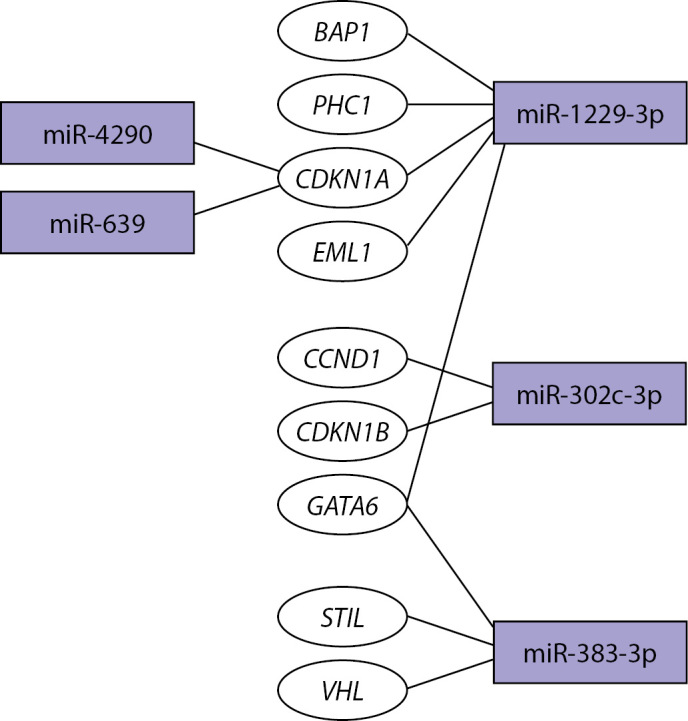
Рисунок 2. Карта взаимодействия микроРНК с генами-мишенями [30].

**Table:** Таблица 7. Характеристика возможных генов-мишеней [41–43][55]

Ген	Описание
CDKN1A	Кодирует ингибитор циклинзависимых киназ, являетсярегулятором клеточного цикла, играет ключевую роль в ответе клетки на повреждение ДНК
GATA6	Кодирует фактор регуляции транскрипции, является онкосупрессором, снижение экспрессии приводит к усилению пролиферации клеток
ZNF264	Кодирует фактор регуляции транскрипции
USP8	Кодирует убиквитин-специфическую протеазу 8. Регулирует содержание внутриклеточных рецепторов EGF в кортикотрофах и влияет на синтез POMC. Активирующая мутация USP 8 является наиболее часто встречающейся в кортикотропиномах
PRKAR1A	Ген регуляторной субъединицы I-альфа. Инактивирующая мутация гена PRKAR1A клинически проявляется формированием Карни-комплекса. Описано всего два случая формирования кортикотропином при Карни-комплексе, гораздо чаще встречается первичная пигментная нодулярная надпочечниковая дисплазия с развитием синдрома Кушинга
CABLES1	CABLES1 повышает экспрессию р27, который, в свою очередь, блокирует клеточную пролиферацию за счет угнетения рецепторов к эпидермальному ростовому фактору (EGF). Инактивирующая мутация потенциально приводит к онкогенному эффекту
CDKN1B	Кодирует ингибитор циклинзависимой киназы 1B р27, являющейся регулятором клеточного цикла (онкосупрессор). Герминальные мутации встречаются у 2٪ пациентов с синдромом, подобным синдрому множественных эндокринных неоплазий
TSC1	Инактивирующая мутация данного гена приводит к формированию туберозного склероза, для которого кортикотропиномы не специфичны, но тем не менее описаны в литературе
SSTR2	Рецепторы соматостатина 2 подтипа
SSTR3	Рецепторы соматостатина 3 подтипа
SSTR5	Рецепторы соматостатина 5 подтипа
TP53	Кодирует один из ключевых опухолевых супрессоров (t53). Мутации данного гена встречаются при неоплазиях разного генеза
VHL	Герминальная инактивирующая мутация приводит в формирования болезни фон Гиппеля–Линдау (VHL-синдром), при котором возможно формирование феохромоцитом и нейроэндокринных опухолей поджелудочной железы, в том числе с секрецией АКТГ и развитием АКТГ-ЭС
SDHC	Мутации данного гена клинически могут проявляться феохромоцитомой и параганглиомой
MAX	Кодирует онкопротеин, участвующий в регуляции клеточного цикла. Клинически мутации в данном гене также могу проявляться феохромоцитомой и параганглиомой
TMEM127	Кодирует трансмембранный протеин 127. Клинически мутации данного гена также описаны при развитии наследственных феохромоцитом и параганглиом
SOX4	Представитель семейства транскрипционных факторов, играющих ключевую роль в эмбриональном развитии гипофиза. Гиперэкспрессия гена SOX4 обнаружена более чем в 20 видах неоплазий, данные многочисленных исследований приводят к выводу, что SOX4 является онкогеном

Далее рассмотрим опубликованные данные касательно выявленных в нашем исследовании микроРНК при других новообразованиях и заболеваниях. В 2018 г. было опубликовано исследование влияния высокого уровня экспрессии длинной некодирующей РНК (lcRNA) HOXC13‐AS на высокие показатели клеточной миграции и пролиферации в тканях назофарингеальных карцином [45]. В ходе исследования было выявлено, что HOXC13‐AS оказывает онкогенное влияние, воздействуя на ось miR-383-3p/HMGA2, а именно снижая уровень экспрессии miR-383-3p и усиливая экспрессию HMGA2. Ранее было выявлено, что miR-383 может играть онкосупрессорную роль в культурах клеток рака молочной железы, подавляя экспрессию Gadd45g (growth arrest and DNA-damage-inducible 45 gamma) [46]. Подавление экспрессии miR-4290 длинной некодирующей РНК DLX6-AS1 связано с ростом клеток рака желудка и обеспечением анаэробного гликолиза по данным исследования Y. Qian [47]. В исследовании 2020 г. отмечено, что высокий уровень экспрессии miR-1229-3p в клетках аденокарциномы желудка и плазме крови пациентов ассоциирован с резистентностью к химиотерапии, в первую очередь к 5-фторурацилу, и потенциально может использоваться при выборе дальнейшей тактики ведения пациентов [48]. В другом исследовании было выявлено, что miR-1229-3p участвует в оси взаимодействия с интегрином-бета 8 (ITGB8), и при ее подавлении кольцевой РНК circ_0037655 наблюдается рост клеток глиомы и увеличивается риск метастазирования [49]. Повышенный уровень экспрессии miR-639 ассоциирован с более тяжелой стадией назофарингеальной карциномы и непосредственно влияет на пролиферацию и миграцию клеток [50]. Изменения в экспрессии miR-639 также выявлены в ткани карцином щитовидной железы: в тканях опухоли уровень экспрессии miR-639 был значительно повышен, ассоциирован с клеточной пролиферацией, предположительно действуя на CDKN1A [51]. Тем не менее, по данным исследования G. Xiao и соавт., miR-639, вероятно, служит онкосупрессором, и ее уровень значительно подавлен в ткани рака печени, в первую очередь посредством гиперметилирования ее промоторного участка гена [52]. В исследовании S. Hamada‐Tsutsumi выявлено противовирусное действие miR-302c-3p на вирус гепатита B, что делает ее потенциальным терапевтическим агентом [53]. В другом исследовании показано, что данная микроРНК является одним из возможных биомаркеров цирроза печени и гепатобилиарной карциномы в исходе гепатита C: уровень ее экспрессии в плазме крови был значительно повышен в указанных группах пациентов по сравнению с группой контроля [54].

Клиническая значимость результатов

Обнаружение панели дифференциально экспрессирующихся микроРНК в периферической крови пациентов с БИК и АКТГ-ЭС смогло бы значительно упростить процесс дифференциальной диагностики АКТГ-зависимых форм ЭГ, убрав необходимость проведения такой инвазивной, дорогостоящей и высокотехнологической процедуры, как селективный забор крови из НКС. Полученные нами данные станут основой для дальнейшей валидизации наиболее отличающихся между группами микроРНК на расширенной выборке пациентов с использованием метода RT-qPCR.

Ограничения исследования

К ограничениям исследования можно отнести малую выборку пациентов и отсутствие группы контроля. Тем не менее следует отметить, что наибольший интерес для нас представляет выявление микроРНК, отличающихся между группами пациентов с БИК и АКТГ-ЭС, так как планируемая в перспективе специфическая панель микроРНК будет использоваться в случаях уже подтвержденного АКТГ-зависимого гиперкортицизма для дифференциальной диагностики. Также следует отметить, что в нашем исследовании мы изучали микроРНК в материале крови от гипофиза, в дальнейших исследованиях необходима валидизация на образцах периферической крови.

Направления дальнейших исследований

Для подтверждения практической значимости микроРНК для дифференциальной диагностики заболеваний, тем более таких редких, как БИК и АКТГ-ЭС, необходимо проводить дополнительные исследования на широкой выборке пациентов. Таким образом, следующим шагом исследования должна стать валидизация полученных данных в периферической крови на расширенной выборке пациентов методом RT-qPCR.

## ЗАКЛЮЧЕНИЕ

В данном исследовании мы продолжили поиск циркулирующих микроРНК, отличающихся между пациентами с БИК и АКТГ-ЭС. По результатам был значительно расширен список микроРНК, потенциально пригодных для формирования панели для малоинвазивной дифференциальной диагностики АКТГ-зависимых форм ЭГ, в первую очередь за счет miR-383-3p, miR-1229-3p, miR-1203, miR-639, miR-4290, miR-6717-5p, miR-302c-3p. Разработка подобной панели значительно упростила бы диагностику тех противоречивых случаев, когда при подтвержденном ЭГ и высоком уровне АКТГ крови не удается обнаружить аденому гипофиза по данным МРТ или ее размеры не превышают 6 мм. Использование более простого и доступного по сравнению с селективным забором крови из НКС метода дифференциальной диагностики позволило бы избежать возможных диагностических ошибок, в том числе проведения заведомо неэффективного нейрохирургического вмешательства пациентам с АКТГ-ЭС.

## ДОПОЛНИТЕЛЬНАЯ ИНФОРМАЦИЯ

Источники финансирования. Исследование выполнено за счет средств гранта РНФ № 19-15-00398.

Конфликт интересов. Авторы декларируют отсутствие явных и потенциальных конфликтов интересов, связанных с содержанием настоящей статьи.

Участие авторов. Белая Ж.Е., Мельниченко Г.А., Малыгина А.А. — концепция и научное руководство исследования; Малыгина А.А, Ситкин И.И., Хандаева П.М., Трухина Д.А. – сбор материала; Никитин А.Г., Кошкин Ф.А. — выделение РНК и анализ экспрессии микроРНК; Никитин А.Г., Малыгина А.А. — статистическая обработка данных; Малыгина А.А.  — написание основного текста рукописи; Белая Ж.Е., Никитин А.Г., Мельниченко Г.А., Луценко А.С., Лапшина А.М. — редактирование текста рукописи. Все авторы внесли значимый вклад в проведение исследования и подготовку статьи, прочли и одобрили финальную версию статьи перед публикацией.
